# Lateral and Medial Ventral Occipitotemporal Regions Interact During the Recognition of Images Revealed from Noise

**DOI:** 10.3389/fnhum.2015.00678

**Published:** 2016-01-06

**Authors:** Barbara Nordhjem, Branislava Ćurčić-Blake, Anne Marthe Meppelink, Remco J. Renken, Bauke M. de Jong, Klaus L. Leenders, Teus van Laar, Frans W. Cornelissen

**Affiliations:** ^1^Laboratory for Experimental Ophthalmology, University Medical Center Groningen, University of GroningenGroningen, Netherlands; ^2^NeuroImaging Center, Department of Neuroscience, University Medical Center Groningen, University of GroningenGroningen, Netherlands; ^3^Department of Neurology, University Medical Center Groningen, University of GroningenGroningen, Netherlands

**Keywords:** DCM, fMRI, object recognition, ventral visual cortex, visual perception

## Abstract

Several studies suggest different functional roles for the medial and the lateral sections of the ventral visual cortex in object recognition. Texture and surface information is processed in medial sections, while shape information is processed in lateral sections. This begs the question whether and how these functionally specialized sections interact with each other and with early visual cortex to facilitate object recognition. In the current research, we set out to answer this question. In an fMRI study, 13 subjects viewed and recognized images of objects and animals that were gradually revealed from noise while their brains were being scanned. We applied dynamic causal modeling (DCM)—a method to characterize network interactions—to determine the modulatory effect of object recognition on a network comprising the primary visual cortex (V1), the lingual gyrus (LG) in medial ventral cortex and the lateral occipital cortex (LO). We found that object recognition modulated the bilateral connectivity between LG and LO. Moreover, the feed-forward connectivity from V1 to LG and LO was modulated, while there was no evidence for feedback from these regions to V1 during object recognition. In particular, the interaction between medial and lateral areas supports a framework in which visual recognition of objects is achieved by networked regions that integrate information on image statistics, scene content and shape—rather than by a single categorically specialized region—within the ventral visual cortex.

## Introduction

Object recognition is a central ability of human visual perception and finding out how the human brain accomplishes it remains an important challenge for vision science. Several studies suggest a distinction between the functional contributions of the more medial and the more lateral sections of the ventral cortex to visual object recognition—with medial sections being more involved in texture processing and lateral sections being more involved in shape processing. Yet, whether and how these medial and lateral sections interact to facilitate object recognition remains largely unknown. Hence, in the present study we asked how object recognition modulates effective connectivity within an occipitotemporal network comprising early visual cortex as well as medial and lateral regions in the ventral cortex. The objective was to investigate whether or not regions in the ventral visual cortex interact during object recognition.

Functional magnetic resonance imaging (fMRI) studies have suggested that the ventral visual cortex consists of specialized “modules” which preferentially respond to specific categories of visual stimuli such as scenes, objects and textures. For instance, the lateral occipital complex (LOC) preferentially responds to objects (Malach et al., 1995; Kanwisher et al., 1997). The LOC can be divided in an anterior part—the posterior fusiform (pFS)—and a posterior part—LO (Grill-Spector et al., 1999). LO has been implicated in physical shape processing and its patterns of activations are more consistent across participants, whereas the pFS has a more perceptually-based representation which varies between participants (Haushofer et al., 2008). Strictly taken, the modular view of the ventral visual cortex does not predict interactions between different regions. Activation in a single region would suffice to achieve recognition. More recently, this modular view has been extended into a network-oriented framework, which suggest that the different regions should interact during visual perception (de Haan and Cowey, 2011; Furl, 2015). However, it is still not clear whether such interactions indeed occur. If we can establish this, we also may be able to determine how the different specialized modules interact within the ventral visual cortex. This, in turn, may provide important clues on how the human visual brain achieves recognition while being faced with the complexity of the natural world.

Dynamic Causal Modeling (DCM; Friston et al., 2003) can provide insight into connectivity and network-properties of visual regions and provide experimental support for models of visual processing (Furl, 2015). This method is particular suited for comparing models involving feed-forward, feedback and reciprocal connectivity between early visual cortex and higher-order regions (i.e., Sterzer et al., 2006; Fairhall and Ishai, 2007). Furthermore, numerous studies have shown interactions between regions within the occipitotemporal cortex during various visual recognition tasks (i.e., Ewbank et al., 2011; Liu et al., 2011; Furl et al., 2015).

Studies on form and texture perception support different roles for the medial and lateral sections of the occipitotemporal cortex (Cant and Goodale, 2007; Cant et al., 2009; Cavina-Pratesi et al., 2010; Park et al., 2011). Moreover, attending to different stimulus properties modulates the recruitment of medial and lateral regions. One study showed that attending to material properties caused an increase of activation in the medial sections of the ventral visual cortex such as the lingual gyrus (LG), the lingual sulcus (LS), and the collateral sulcus (CoS) (Cant and Goodale, 2007). Similar patterns emerge in other studies. Medial regions comprising the LG and CoS were involved in texture discrimination, while shape discrimination modulated activation in the LOC (Peuskens et al., 2004). Varying either the shape or the texture of objects activated lateral or medial sections of the ventral cortex, respectively, (Cavina-Pratesi et al., 2010).

In further support, patient studies suggest a double dissociation between processing of shape and material properties. Patients with damage to the lateral sections of the ventral visual cortex are unable to perceive the form and shape of objects (visual form agnosia), while they can still perceive their texture and color (James et al., 2003). The opposite is seen in patients with damage to the medial ventral cortex. These patients are unable to perceive color but can still perceive form (cerebral achromatopsia; for a review see Heywood and Kentridge, 2003).

Finally, results from our own group (Meppelink et al., 2009) also point toward a specific role for medial sections of the ventral cortex in object recognition. For images gradually being revealed from noise, we found an increase of neural activity in the LG at the moment of recognition. This contrasts with the classical view that proposes LOC as the primary region for object recognition. Taken together, there is reason to ask whether and how these medial and lateral sections within the ventral visual cortex interact to facilitate object recognition.

In the current study, we investigated the effective connectivity between medial and lateral occipito-temporal sections of the ventral visual cortex during the recognition of images. Normally, object recognition takes places within a fraction of a second. However, we used a stimulus for which the process of recognition was extended over time. Observers had to recognize images containing objects that were gradually revealed from a background of visual noise. The observers indicated when an object was recognized. This allowed us to include and compare both the period before and after recognition in our analysis. Hence, we asked how object recognition modulates effective connectivity within an occipito-temporal network.

Determining functional connectivity requires selecting a number of target regions of interest (ROIs). We focused on how a network comprising the primary visual cortex (V1), a medial and a lateral section of the ventral visual cortex interacts during object recognition. We choose the lingual gyrus (LG) as the medial section of the network, based on its involvement in texture and scene processing, as well as during pop-out (Meppelink et al., 2009). As the lateral section, we choose the lateral occipital cortex (LO), based on its involvement in object recognition (Grill-Spector et al., 1999). Our aim was to investigate the dynamic relationships between V1, a medial, and a lateral section of the ventral visual pathway during object recognition. Hence, within each hemisphere, we defined V1 as a ROI, and included LO as the lateral ROI, and LG as the medial ROI. Using DCM, we sought to elucidate whether the various connections in this network are characterized by feed-forward, feedback, or a bi-directional architecture.

## Materials and methods

We used fMRI data—collected in a previous study (Meppelink et al., 2009)—in which subjects recognized images of objects and animals that were gradually revealed from noise. Object recognition is a very rapid process and the underlying mechanisms can be difficult to disentangle with fMRI due to its relatively low temporal resolution. To investigate the dynamic processes involved in object recognition with fMRI, the study was done with images that were gradually revealed from random noise. Breaking up the process of recognition has been shown to give a more detailed picture of activation in the brain before and after recognition (James et al., 2000; Kleinschmidt et al., 2002; Reinders et al., 2006). The slow appearance of the images allowed us to compare pre- and post-recognition of the stimuli by prolonging the period before recognition. The fMRI results have been reported in detail elsewhere (Meppelink et al., 2009). The original study included both patients and healthy controls, but for our present purpose, only the data from the healthy subjects was analyzed. We summarize the experimental setup (details can be found in Meppelink et al., 2009). Following this, the DCM analysis is explained.

### Participants

Fourteen healthy participants (mean age 58.5, SD 7.5, range 47–71, four males) participated. Visual acuity was assessed with the Snellen chart. Exclusion criteria were dementia (MMSE score < 24), neurological disorders, psychiatric disorders, visual acuity < 50% (Snellen chart), and visual field defects. One participant was excluded due to excessive motion artifacts.

### Ethics statement

This study was approved by the Medical Ethical Committee of the University Medical Center Groningen. All participants signed an informed consent prior to the study. Participants were informed that the experiment was voluntary and they could terminate their participation at any time.

### Stimuli and experimental paradigm

Stimuli consisted of 50 gray-scale pictures of animals (22), well-known objects (22), and meaningless objects (6). The images had a resolution of 300 × 300 pixels and the movies were scaled to twice this size. Images were first normalized to have their mean luminance equal to the background level, and were gradually revealed from random uniform visual white noise in movie sequences with a duration of 30 s. The noise contrast remained the same throughout the movies while the image contrast increased gradually over time. This increase of signal-to-noise made the image appear to “pop-out.” Movie stimuli were generated in Matlab 5 augmented with routines from the Psychtoolbox (Brainard, 1997; Pelli, 1997). The movies were presented using Presentation (Neuro Behavioural Systems, Inc., CA, USA). Movies were presented in two runs, with 25 movies per run. Each movie sequence was only shown once. Object recognition was indicated by key-presses. To control for reaction time and keep the participants attending, they performed an additional task. A central fixation point changed color with random intervals throughout the experiment. Participants were asked to report the color changes by pressing a second key. Pop-out occurred between 10 and 28 s after initial movie onset. The experiment also included a separate localizer session. The classical localizer was used to guide the localization of LO for the connectivity analysis. The localizer stimuli consisted of intact and block scrambled (20 × 20) images of objects and animals. The images were shown in 15 s sequences of gray-scale images alternating with 15 s sequences of scrambled versions of the same images, with 15 s between each sequence. Images were displayed for 3 s each. Subjects were instructed to passively view the stimuli.

### Data acquisition

Data were acquired with a 3 Tesla Philips MR system (Best, The Netherlands) with a standard six-channel SENSE head coil [echo time (TE) 35 ms, repetition time (TR) of 2.3 s, 35 slices per TR, 450 volumes per run] ascending order with isotropic voxels size of 3 × 3 × 3 mm^3^ and an axial orientation. A T1 weighted anatomical scan with 1 × 1 × 1 mm^3^ isotropic voxels was acquired for high-resolution anatomical information, matrix size = 256 × 256 and an axial orientation.

### Voxel-based analysis

**Figure 1:**
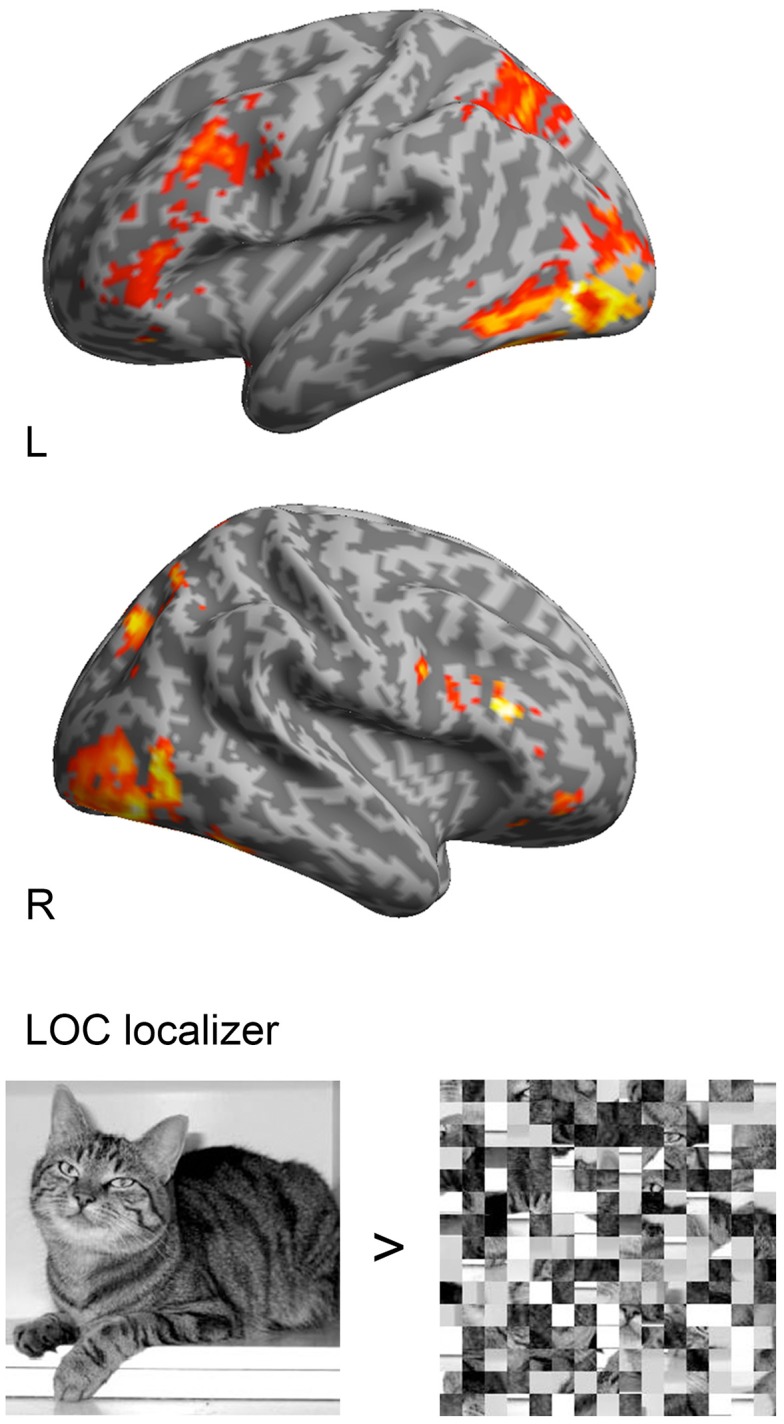
**Group analysis (***n*** = 14) of the LOC localizer (objects > scrambled objects) with a threshold of ***p*** < 0.05, FSW corrected and superimposed onto a standard 3D inflated template in MNI space**. Activations were found in lateral occipital cortex with the MNI coordinates 48, −54, −9 and −45, −54, −15.

**Figure 2:**
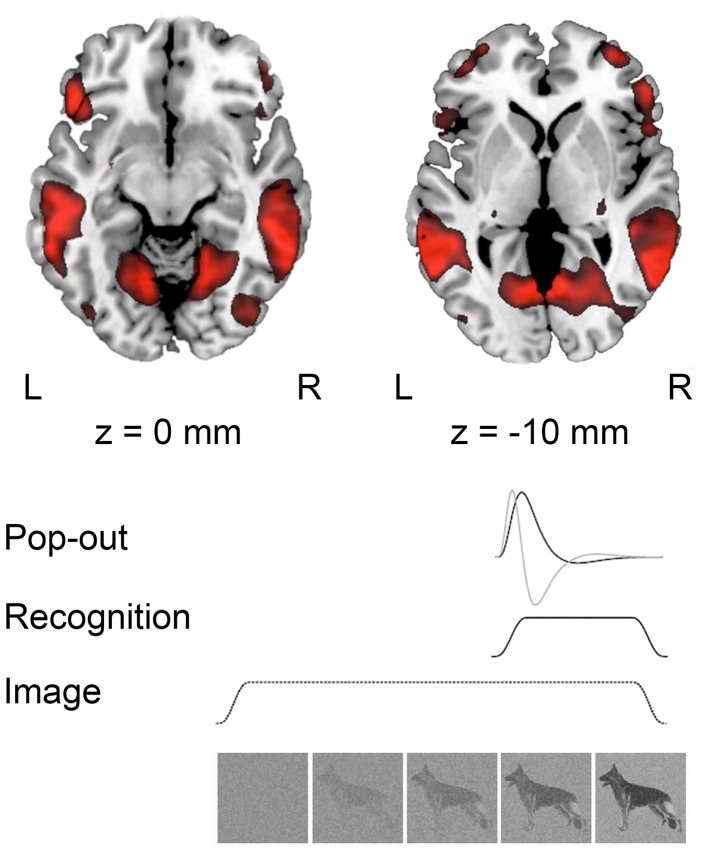
**Group activations (***n*** = 13) during recognition at ***p*** < 0.001, uncorrected projected on a standard template in MNI space**. Below a schematic representation of the modeled responses during image presentation; “Pop-out” indicating the moment of recognition, “Recognition” modeled from pop-out to the end of the trial and “Image” modeling the whole trial.

Data were analyzed in SPM 8 (Wellcome Department of Imaging Neuroscience, London; http://www.fil.ion.ucl.ac.uk/spm). Pre-processing included realignment, slice time correction, spatial normalization (to the EPI template of the Montreal Neurological Institute, MNI), and smoothing with a Gaussian filter of 8 mm full width at half maximum (FWHM). Following preprocessing, the data was entered into a general linear model. The regressors for the localizer scan (Figure 1) and recognition task (Figure 2) are described in the following section and more details on the analysis (Meppelink et al., 2009). All regressors were convolved with the canonical hemodynamic response function.

The moments of recognition were time-locked on the perceptual pop-out and modeled as a stick-function and the time derivative. We also modeled the block of visual recognition, lasting from the moment of pop-out to the end of the trial, as well as a 30 s block for the full trial periods of visual input. Hence, the design matrix included the moment of “pop-out,” “recognition” from the moment that pop-out was indicated to the end of the trial, and “image” modeling the whole trial. Movement parameters were included as covariates.

The localizer, which was used to delineate LOC, included regressors for intact and scrambled objects. T-contrasts for intact objects compared to scrambled objects were made for each subject. Individual contrast images were entered into random-effects analyses at the second level (one-sample *t*-tests). Activations in the random-effects analyses were considered significant at *p* < 0.05 (FWE corrected).

### Effective connectivity

DCM allows an assessment of the connectivity between cortical regions. DCM is suitable to estimate interactions between brain regions on the neuronal level and the degree by which these interactions are affected by experimental perturbations (Stephan et al., 2010). We used DCM to test how object recognition modulated effective connectivity within a network consisting of ROIs in V1, LG, and LO within each hemisphere. DCM describes the neuronal interaction in the form of the bilinear state equation where the neural dynamics during experimental manipulation is modeled by differential equations (Friston et al., 2003). Three sets of parameters are estimated with DCM: the external influence of inputs on regions of interest, the intrinsic connections between regions without the experimental manipulation, and the modulation of the connections induced by the experimental condition (Friston et al., 2003). The advantage of DCM upon other effective connectivity techniques is that it incorporates the hemodynamic balloon model in order to estimate the actual neuronal dynamics from measured fMRI data. In the current analysis, we were specifically interested in the modulatory effect of object recognition from the moment of recognition (“pop-out”) to the end of the trial (hereafter referred to as “the modulatory effect of recognition”). The DCM analysis was carried out with SPM8 (www.fil.ion.ucl.ac.uk/spm) using DCM 10. We performed the DCM analysis in several steps (Penny et al., 2004). First, time series were extracted from various ROIs (see Section ROI Selection and Time Series Extraction for details). Second, 64 possible models were created and estimated for each subject (see Section Model Space for details). Third, we compared these 64 models across the 13 subjects using Bayesian model selection (BMS) in order to determine the most likely model (see Section Bayesian Model Selection and Statistics for Details). Finally, one-sample *t*-tests were made for the parameter estimates.

### ROI selection and time series extraction

We modeled a network including V1, LG, and LO based on functional and anatomical constraints within each hemisphere. The peak coordinates from the group analysis (Table 1) were used to determine to determine the region where we would define subject-specific ROIs and extract time-courses. Then we looked for subject-specific activation in as close proximity as possible to the group results to define each ROI. The SPM Anatomy toolbox was further used to guide the ROI location for each subject (Eickhoff et al., 2005). This approach ensures that time series for each subject are both functionally and anatomically standardized (Stephan et al., 2007a).

**Table 1:** **Guiding voxels for time series extraction**.

**Paradigm**	**Anatomical region**	**MNI coordinates**		
		***x***	***y***	***z***
Pop-out	Left V1	−6	−95	0
	Right V1	12	−92	5
	Left Lingual gyrus	−21	−54	0
	Right Lingual gyrus	18	−42	0
LOC Localizer	Left Inferior occipital	−42	−54	−15
	Right Inferior occipital	48	−54	−9

*MNI, Montreal Neurological Institute, units are in millimeters*.

We extracted individual time series from the pop-out experiment, with contrasts made at a threshold of *p* < 0.001, uncorrected. We used two different contrasts for each subject to extract the time series from the pop-out experiment. To identify V1, we compared whole blocks of visual stimulation for each subject. Within each hemisphere, V1 was identified by a local maximum within the calcarine sulcus, located within BA 17 determined by the SPM Anatomy Toolbox (Eickhoff et al., 2005). We extracted the time series for both LG and LO by contrasting recognition (pop-out to the end of each trial) with baseline. We used group peak coordinates from the localizer to guide the ROI extraction of LO (Table 1). In the main recognition experiment, there was activation in LG during recognition; these coordinates were used to guide the ROI extraction of LG (Table 1). The centers of the ROIs for each subject were selected within a radius of 16 mm of the guiding voxel and belonging to the same anatomical region. We defined a 6 mm sphere around each center and extracted the time series within this region. The first eigenvariate was computed for voxels within the sphere and used for further analysis. Time series were extracted separately for each session and adjusted for effects of interest. Mean coordinates of voxels representing the center of ROIs and the standard deviations from these coordinates are listed in Table 2.

**Table 2:** **Mean coordinates (and the standard deviation) for the ROIs**.

**Anatomical region**	**Left MNI coordinates**			**Right MNI coordinates**		
	***x***	***y***	***z***	***x***	***y***	***z***
V1	−13(5)	−96(3)	−4(4)	17(3)	−95(2)	−2(4)
Lingual gyrus	−14(6)	−67(6)	−6(6)	18(5)	−63(10)	−5(6)
Lateral occipital	−51(8)	−64(14)	−7(6)	52(7)	−63(10)	11(4)

*MNI, Montreal Neurological Institute, units are in millimeters*.

### Model space

**Figure 3:**
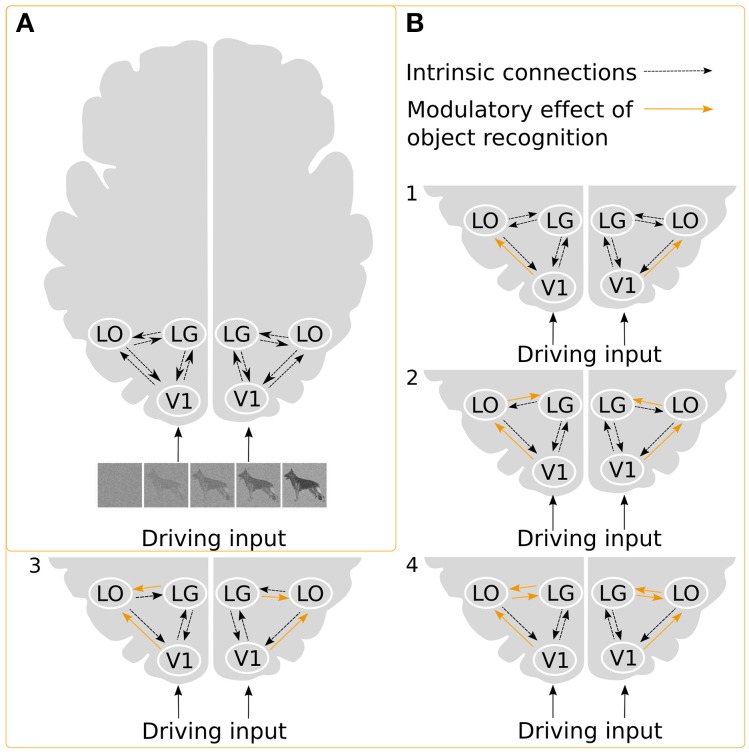
**Illustration of the DCMs**. **(A)** Intrinsic connections and the driving input of the stimuli. **(B)** Examples of possible ways in which object recognition could modulate effective connectivity. In model 1, object recognition alters the connectivity from V1 to LO, and modulation of the intrinsic connection changes between LG and LO, in model 2, object recognition modulates connectivity from V1 to LO as well as from LO to LG, while in model 3, the connectivity from LG to LO is modulated. Finally, in model 4, modulations affect both directions between LG and LO.

We constructed a basic model with reciprocal intrinsic connections between all three ROIs for each hemisphere. We choose to have bidirectional intrinsic connections between all ROIs within each hemisphere due to the highly interconnected nature of the visual cortex (Kravitz et al., 2013). The regressor describing the whole image sequence was defined as the driving input. We assumed that the driving input would enter the model from V1. To explore the modulatory effect of recognition, we created a model space consisting of all possible combinations, thus including modulatory effects on forward, backward and reciprocal connections (Figure 3). Based on these choices, we could build 2^6^ = 64 models of the modulatory effect of recognition.

### Bayesian model selection and statistics

For each subject, all candidate models were estimated. Following this, the 64 models were compared in a pairwise fashion using the Bayesian Model Selection tool (BMS) (Penny et al., 2004; Stephan et al., 2007b, 2009) at the group level. We used a random-effects (RFX) analysis for the group level analysis because it takes the heterogeneity of models across subjects into account, while a fixed effect (FFX) analysis is more vulnerable to outliers (Stephan et al., 2007b). Generally, RFX is considered better suited for modeling cognitive tasks because subjects may have different winning models (Stephan et al., 2010). The RFX results are reported in terms of exceedance probabilities (probability that the models outperforms others) and expected posterior probability (the likelihood of obtaining the given model for a randomly selected subject from the population). Following, one-sample *t*-tests were computed to assess if the individual parameters deviated from zero. The *t*-tests were carried out across participants for each parameter of the intrinsic connections as well as on the modulatory connections of the winning model. Parameter values were considered significantly different from zero at *p* < 0.05 correcting for multiple comparisons using false discovery rate (FDR).

## Results

### fMRI results

In the analysis of the localizer scan, activation during the presentation of intact and scrambled objects was contrasted. As expected, we found activation in the LOC (Figure 1 and Table 3A). Within each hemisphere, two peaks of activation were identified, corresponding to LO in the lateral occipital cortex and the posterior fusiform gyrus. Further, we contrasted activation before and after the moment of recognition in the pop-out experiment (Figure 2, Table 3B). The period before recognition was characterized by activation in early visual areas located in the posterior occipital lobe. The results revealed bilateral frontal and temporal activations during the period after recognition. Also, activations were found in the left inferior parietal lobe, the right lingual gyrus and calcarine gyrus and the left cuneus.

**Table 3:** **(A) LOC localizer to guide the identification of LO by contrasting unscrambled with scrambled objects, (B) regions of cerebral activations before (Image > Recognition) and after the moment of recognition (Recognition > Image)**.

**Contrast/region**	**Localization**	**Hemisphere**	**MNI Coordinates (mm)**			***Z***
			***x***	***y***	***z***	
**(A) LOC LOCALIZER**						
**Unscrambled** > **scrambled**						
Temporal	Fusiform gyrus	L	−42	−54	−15	5.43
	Inferior temporal gyrus	R	48	−54	−9	5.62
**(B) POP-OUT EXPERIMENT**						
**Before recognition**						
Occipital	Middle occipital gyrus	L	−24	−93	0	5.37
	Calcarine sulcus	R	15	−96	0	5.5
**After recognition**						
Frontal	Middle Frontal Gyrus	L	−24	45	30	5.55
	Superior Medial Gyrus	L	−9	39	42	5.53
	Medial Frontal Gyrus	R	36	51	9	4.24
Parietal	Inferior parietal lobe	L	−42	−60	24	5.69
Temporal	Middle Temporal Gyrus	L	−60	−30	−3	5.33
		R	54	−39	−3	4.55
Occipital	Cuneus	L	−3	−78	30	5.32
	Lingual Gyrus	R	−6	−54	0	5.52
	Calcarine Gyrus	R	12	−75	15	5.82

*MNI, Montreal Neurological Institute; L, left, R, right. Reported regions were significant at a cluster threshold of p < 0.001 FWE corrected or a peak threshold, p < 0.05, FWE corrected*.

### Effective connectivity and modulatory effects

**Figure 4:**
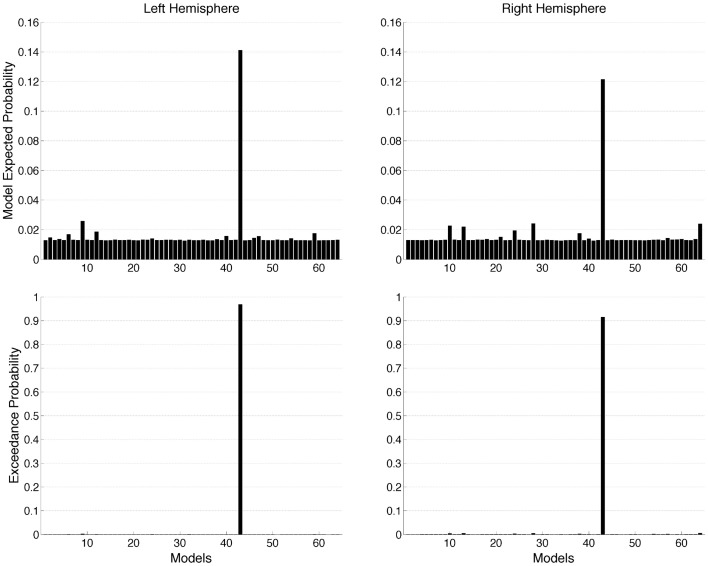
**Random (RFX) effects Bayesian model selection (BMS) at group level estimated for 64 models**. The graphs show model expected probability and model exceedance probability. Model 43 outperformed all other models in both hemispheres.

We used DCM to investigate the effective connectivity in a network consisting of V1, LG, and LO in both the left and the right hemisphere. We compared 64 models of all possible different combinations of effective connections. The driving input was the same for all models. The modulatory effect of object recognition was modeled for all the effective connectivities. The results of the group Bayesian model selection (BMS) showed that for both hemispheres one model (M43) clearly outperformed all others [exceedance probability of 0.97 and 0.92 (*n* = 13) for the left and right hemisphere, respectively, (Figure 4)]. The winning model indicates that for both hemispheres, object recognition modulated connectivity from V1 to both LG and LO as well as the bidirectional connectivity between LG and LO. This winning model was found most likely in 9 of the 13 subjects for the left hemisphere and in 8 of the 13 subjects for the right hemisphere.

**Figure 5:**
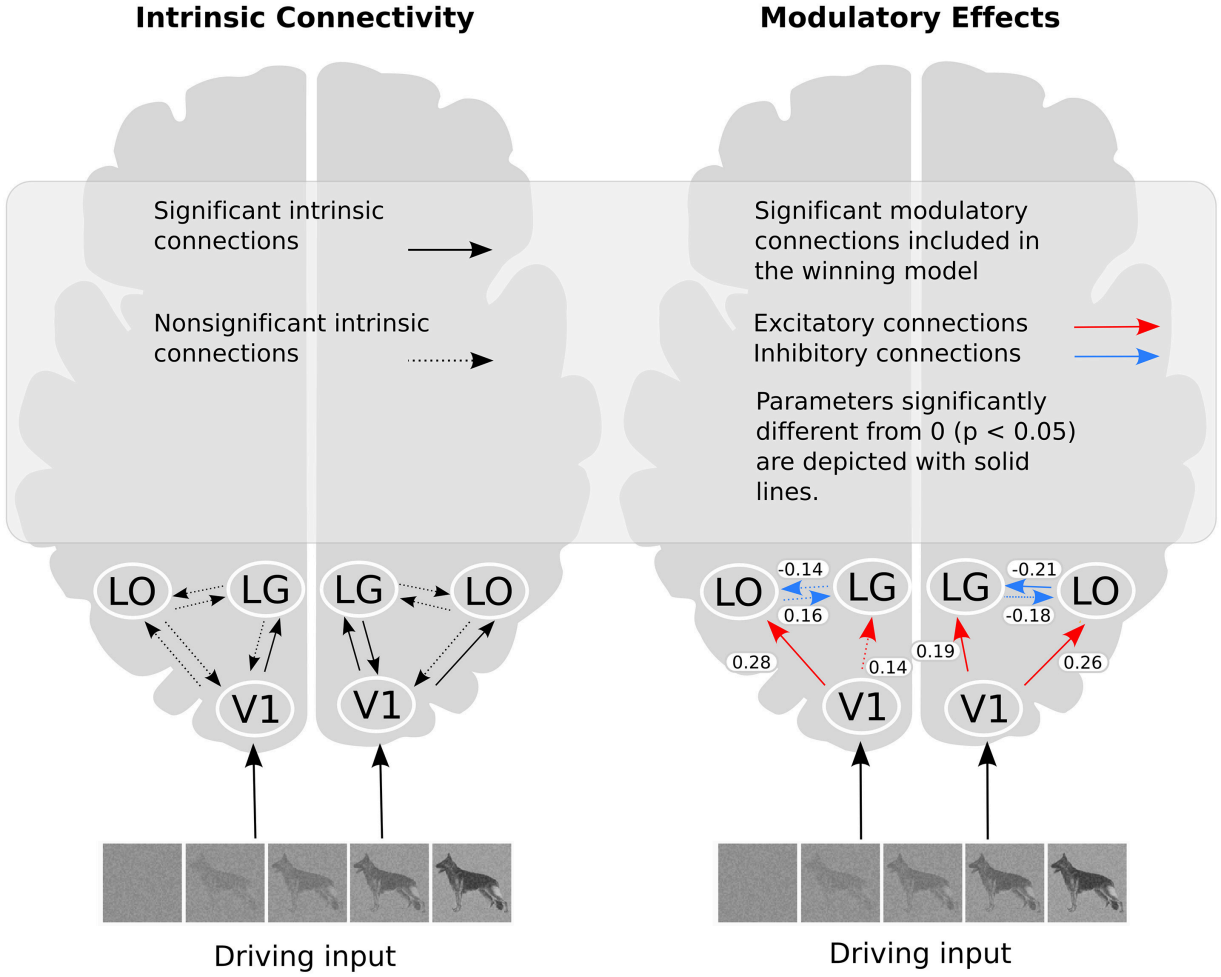
**The winning model and the modulatory effect of recognition**. In the right part of the figure, the values shown refer to the average parameter estimates.

The posterior parameter estimates averaged across subjects are depicted in Figure 5 and listed in Table 4. For completeness, both intrinsic and modulatory connections are shown. One-sample *t*-tests were performed to determine if individual posterior parameter estimates differed from 0 with the threshold for statistical significance set at *p* < 0.05 correcting for multiple comparisons using false discovery rate (FDR). In the left hemisphere, average posterior parameter estimation showed that the connection from V1 to LO was positive and differed from zero. This indicates that V1 activity enhances the activity in LO during recognition. For the left hemisphere, no other posterior parameter estimates reached significance. In the right hemisphere, object recognition significantly modulated the connectivity from V1 to LO and V1 to LG. Posterior parameter estimates for both connections were significantly larger than zero, indicating that V1 activity enhanced the activity in both LG and LO during recognition. In addition, in this hemisphere, the modulatory influence of LO on LG was significantly below zero, indicating suppression of activity. The modulatory influence of LG on LO did not reach significance.

**Table 4:** **Coefficient means and standard deviation for the modulations of the connections in the winning model**.

**Connection**	**Coefficient mean**	**Standard deviation**
**INTRINSIC**		
**Left**		
**V1 → LG**	−**0.18**	**0.11**
V1 → LO	0.08	0.18
LG → LO	−0.07	0.22
LO → LG	0.26	0.74
LG → V1	0.54	1.28
LO → V1	−0.02	1.36
**Right**		
**V1 → LG**	−**0.12**	**0.09**
**V1 → LO**	**0.15**	**0.11**
LG → LO	0.24	0.41
LO → LG	−0.09	0.33
**LG → V1**	**0.79**	**1.10**
LO → V1	−0.46	1.04
**MODULATORY**		
**Left**		
V1 → LG	0.14	0.30
**V1 → LO**	**0.28**	**0.23**
LG → LO	−0.14	0.52
LO → LG	−0.16	0.75
**Right**		
**V1 → LG**	**0.19**	**0.29**
**V1 → LO**	**0.26**	**0.25**
LG → LO	−0.18	0.46
**LO → LG**	−**0.21**	**0.20**

*Modulations were considered significantly different from zero at p < 0.05 (FDR corrected). Connections with significant modulations are shown in bold*.

## Discussion

In this study, we investigated the modulation of functional connections in the ventral visual cortex during the recognition of images that were gradually revealed from noise. We focused on the modulatory effect of recognition on a small occipitotemporal network comprising V1, the Lingual Gyrus (LG) and the Lateral Occipital (LO) cortex. Using DCM, we found that recognition reciprocally altered the effective connectivity between LG and LO. In addition, the feed-forward—but not the feedback—connectivity from V1 to LG and LO was modulated. These findings support the view that visual object recognition is accomplished by networked areas that integrate information on image statistics, texture, scene content and shape—rather than by a single categorically specialized region—within the ventral visual cortex. Below, we discuss our findings in more detail.

### Object recognition reciprocally modulates the connectivity between medial and lateral regions

Bayesian model selection (BMS) shows which model is most likely to explain the data. It indicated that a model comprising bidirectional coupling between LG and LO provided the best explanation for the changes in effective connectivity during objection recognition in both the left and right hemisphere. The finding is in line with a distributed view of recognition, involving networked brain areas (e.g., de Haan and Cowey, 2011) and corroborates other fMRI studies supporting different but complimentary roles of medial and lateral regions (Park et al., 2011). The reciprocal modulation of connectivity between LG and LO could reflect integration of complementary information processing carried out in each region, such as texture and shape. Medial sections of the ventral cortex have been linked with surface and texture processing while LO has been linked with form processing (Cant et al., 2009). Additionally, modulation of connectivity between LG and LO may relate to eccentricity-based differences in processing in which lateral sections of the ventral visual cortex respond more strongly to foveal object information while medial sections are biased toward objects in the peripheral visual field (Levy et al., 2001). We note that these biases may be related: coarse, texture-based processing which relies mainly on peripheral vision could be supported by medial regions, while shape and finer detail which relies on foveal vision could be processed in more lateral sections of the ventral visual cortex. However, such interpretations remain speculative. Overall, the reciprocal modulation of the connections between the two higher-order visual areas revealed by our study suggests that such lateral connections play an integral role in object recognition. The interaction between medial and lateral areas supports that visual recognition of objects is achieved by network that integrates image statistics and scene content (Oliva et al., 2001; Greene and Oliva, 2009) as well as shape information.

### Object recognition modulates the feed-forward but not the feedback connectivity from V1 to both the medial and lateral sections of the ventral cortex

Object recognition altered the effective connectivity from V1 to LG and LO in both hemispheres. This implies that information for object recognition is transferred in parallel from V1 to both the medial (LG) and the lateral (LO) sections. The modulation of the feed-forward connections presumably reflects the activation of specific feature-filters that extract texture, image statistics or shape information from V1-derived information in the two ventral regions.

At the same time, the winning model implies that object recognition did not modulate the feedback connectivity from LG and LO back to V1. This finding contrasts frameworks that propose that feedback from higher to lower areas is essential for object recognition (Lamme et al., 1998). Top-down influence is also highlighted in the Reverse Hierarchy Theory (RHT; Ahissar and Hochstein, 2000) where high-level representations are projected backwards and modulate early visual regions. On the one hand, it is possible that our task simply did not require feedback to V1 to achieve recognition. On the other hand, we should note that our result does not rule out the existence of feedback from LG and LO to V1. It is possible that such feedback was continuously present and not specifically modulated by recognition. Feedback may be related to high-level processes such as selective visual attention. For instance, the RHT is specifically concerned with spatial attention and target detection tasks. Also, there is evidence for modulation of V1 based on high-level interpretations of ambiguous stimuli (Hsieh et al., 2010). It is possible that in situations where one has to attend to certain features while ignoring others, more feedback related activity would occur. Such processes may not have been engaged by our task but could be identified with different stimuli or tasks.

### Individual connections

Bayesian model selection (BMS) shows which model is most probable given the data. It indicated that a single model provided the best explanation for the changes in effective connectivity during objection recognition in both the left and right hemisphere. The winning model incorporates connectivity from V1 to LG and LO as well as a bidirectional coupling between LG and LO.

However, BMS cannot be used to make inferences at the level of the individual connections. Therefore, individual connectivity parameters of the winning model were evaluated by performing one-sample *t*-tests across subjects. In the left hemisphere, one of the four connections reached significance and in the right hemisphere three out of four connections reached significance by themselves. The non-significant connections most likely reflect individual differences amongst subjects. Therefore, in our conclusions and discussion, we focus on the implications of the winning model and do not draw strong conclusions based on the individual parameters.

Nevertheless, it is worth to examine the possibly implications of the individual connections that did reach significance. In both hemispheres, the connections originating from V1 were positive, which indicates that modulation from V1 exerted an excitatory effect on LG and LO. In the right hemisphere, the forward connections from V1 to LG and from V1 to LO were significant, while in the left hemisphere the connection from V1 to LO was significant.

In the right hemisphere, the connection from LO to LG was negative indicating it was inhibitory in nature. This inhibition of LG by LO could imply that these regions are competing, an interpretation consistent with biased competition models which suggest that neurons selective for different visual properties inhibit each other in the presence of their preferred stimulus (Desimone and Duncan, 1995; Reynolds and Chelazzi, 2004). None of the other three lateral connections reached significance. This indicates variability in the nature of the modulations (i.e., in whether they were positive or negative). In turn, this may reflect individual differences in how observers “solved” the object recognition problem (e.g., in whether they were more inclined to base their decision on texture statistics or on shapes and contours). However, without further evidence to select or weigh each observer's contribution, our present study does not allow us to further investigate this option.

### Limitations

The number of participants in this study was thirteen, which is not very high. While the DCM analysis clearly selected a winning model, the number of participants may have limited our ability to draw conclusions regarding the individual connection strengths.

Recent studies have also shown that knowledge of anatomical connections for each participant can improve the DCM analysis by adding priors based on tractography to the model (Stephan et al., 2009). As we did not have such information available for our participants, this study can only address effective connectivity. However, it is important to note that functional and effective connectivity is not fully determined by anatomical connections. The correspondence between anatomical connections and effective connectivity need not be complete. Numerous studies of neural dynamic networks during resting-state support that functional integration is dynamic (i.e., Ghosh et al., 2008). Such dynamic properties of the brain could rely on short term plasticity and neuromodulation (Zucker and Regehr, 2002; Montgomery and Madison, 2004).

The current study was limited to a network consisting of three regions. We selected these regions for reasons mentioned in the introduction. At the same time, we are aware that this number does not represent the full complexity of the neuronal architecture underlying object recognition. For example, anatomically, back projections can be found between almost all regions of the ventral cortex (Felleman and Van Essen, 1991). Hence, it is likely that the regions in our small network received input and feedback from other regions than only those included in the current model. Future studies could investigate if there is top-down modulation from higher-order brain areas that influence this network.

## Conclusion

Using DCM, we investigated connectivity in an occipital-temporal network during the recognition of images gradually revealed from noise. Recognition modulated the feed-forward connections from V1 to both LG and LO, but not feedback connections between these regions and V1. The modulation of the feed-forward connections presumably reflects the activation of specific feature-filters for texture, image statistics or shape in ventral regions. In addition, the bidirectional coupling between LG and LO implies that reciprocal connections between medial and lateral sections of the ventral visual cortex are important to achieve successful recognition. In particular, this interaction between medial and lateral areas supports a framework in which visual recognition of objects is achieved by networked regions that integrate information on image statistics, scene content, and shape—rather than by a single categorically specialized region—within the ventral visual cortex.

### Conflict of interest statement

The authors declare that the research was conducted in the absence of any commercial or financial relationships that could be construed as a potential conflict of interest.
